# Individualized prediction of mortality using multiple inflammatory markers in patients on dialysis

**DOI:** 10.1371/journal.pone.0193511

**Published:** 2018-03-01

**Authors:** Hee-Yeon Jung, Su Hee Kim, Hye Min Jang, Sukyung Lee, Yon Su Kim, Shin-Wook Kang, Chul Woo Yang, Nam-Ho Kim, Ji-Young Choi, Jang-Hee Cho, Chan-Duck Kim, Sun-Hee Park, Yong-Lim Kim

**Affiliations:** 1 Department of Internal Medicine, Kyungpook National University Hospital and School of Medicine, Kyungpook National University, Daegu, Korea; 2 Clinical Research Center for End Stage Renal Disease, Daegu, Korea; 3 Department of Statistics, Kyungpook National University, Daegu, Korea; 4 Department of Internal Medicine, Seoul National University College of Medicine, Seoul, Korea; 5 Department of Internal Medicine, Yonsei University College of Medicine, Seoul, Korea; 6 Department of Internal Medicine, The Catholic University of Korea College of Medicine, Seoul, Korea; 7 Department of Internal Medicine, Chonnam National University Medical School, Gwangju, Korea; 8 Bk21 Plus KNU Biomedical Convergence Program, Department of Biomedical Science, Kyungpook National University, Daegu, Korea; Kaohsiung Medical University Hospital, TAIWAN

## Abstract

This study aimed to evaluate whether the combination of inflammatory markers could provide predictive powers for mortality in individual patients on dialysis and develop a predictive model for mortality according to dialysis modality. Data for inflammatory markers were obtained at the time of enrollment from 3,309 patients on dialysis from a prospective multicenter cohort. Net reclassification index (NRI) and integrated discrimination improvement (IDI) were calculated. Cox proportional hazards regression analysis was used to derive a prediction model of mortality and the integrated area under the curve (iAUC) was calculated to compare the predictive accuracy of the models. The incremental additions of albumin, high-sensitive C-reactive protein (hsCRP), white blood count (WBC), and ferritin to the conventional risk factors showed the highest predictive powers for all-cause mortality in the entire population (NRI, 21.0; IDI, 0.045) and patients on peritoneal dialysis (NRI, 25.7; IDI, 0.061). The addition of albumin and hsCRP to the conventional risk factors markedly increased predictive powers for all-cause mortality in HD patients (NRI, 19.0; IDI, 0.035). The prediction model for all-cause mortality using conventional risk factors and combination of inflammatory markers with highest NRI value (iAUC, 0.741; 95% CI, 0.722–0.761) was the most accurate in the entire population compared with a model including conventional risk factors alone (iAUC, 0.719; 95% CI, 0.700–0.738) or model including only significant conventional risk factors and inflammatory markers (iAUC, 0.734; 95% CI, 0.714–0.754). Using multiple inflammatory markers practically available in a clinic can provide higher predictive power for all-cause mortality in patients on dialysis. The predictive model for mortality based on combinations of inflammatory markers enables a stratified risk assessment. However, the optimal combination for the predictive model was different in each dialysis modality.

## Introduction

Although there have been recent advances in management of end-stage renal disease (ESRD), mortality rates in patients with ESRD remain high [1]. Therefore, early risk stratification and prompt therapy of patients at high mortality risk are crucial elements of ESRD care. Traditionally, Framingham risk factors were considered as accurate predictors for coronary heart disease in the general population [2]. Considering that the risks for patients on dialysis are different from other populations, conventional risk factors for mortality cannot be simply applied to patients with ESRD. Multiple studies have attempted to discover additional biomarkers, which improve the predictive power of mortality beyond the established risk factors in patients on dialysis [3]. Emerging biomarkers for mortality in patients with ESRD consist of cardiac biomarkers [4–6], vascular calcification biomarkers, and inflammatory biomarkers [3]. We studied the additional predictive powers of inflammatory biomarkers above those of conventional risk factors.

Chronic inflammation has been recognized as a major pathophysiologic phenomenon in patients with ESRD. Considering the role of chronic inflammation on mortality of patients with ESRD, attempts to identify patients at greatest mortality risk and to stratify risk of mortality based on inflammatory markers is essential. Although numerous studies have demonstrated the significant association between inflammatory markers, such as serum albumin [7,8], C-reactive protein (CRP) [9–11], white blood cell count (WBC) [12,13], ferritin [14–16], and inflammatory cytokines [17,18], and mortality in patients on dialysis, these results do not quantify the improvement in risk prediction of adding these inflammatory markers. It is known that weak or moderate relationships between biomarkers and outcome can be statistically significant if examined using a large sample size [19]. Therefore, the net reclassification improvement (NRI) and the integrated discrimination improvement (IDI) could better address the added risk predictive ability of biomarkers beyond the established risk models. NRI evaluates the ability of a biomarker to discriminate subjects who will develop an event and who will not. IDI focuses on improvement of average sensitivity, without sacrificing specificity, for models with and without the biomarkers [19].

This study aimed to investigate 1) whether multiple inflammatory markers measured in routine clinical practice could improve risk prediction for mortality in patients on dialysis, using reclassification and discrimination analyses, and 2) whether the predictive model is different in each dialysis modality.

## Materials and methods

### Patients

A total of 3,309 patients, age 19 years or older, who initiated or continued dialysis due to ESRD, were enrolled from a prospective multicenter cohort study in Korea (Clinical Research Center for End Stage Renal Disease, CRC for ESRD, clinicaltrial.gov NCT00931970). All patients provided their written informed consent before participating in the study and the Institutional Review Board of each center approved the study protocol. [The Catholic University of Korea, Bucheon St. Mary’s Hospital; The Catholic University of Korea, Incheon St. Mary’s Hospital; The Catholic University of Korea, Seoul St. Mary’s Hospital; The Catholic University of Korea, St. Mary’s Hospital; The Catholic University of Korea, St. Vincent’s Hospital; The Catholic University of Korea, Uijeongbu St. Mary’s Hospital; Cheju Halla General Hospital; Chonbuk National University Hospital; Chonnam National University Hospital; Chung-Ang University Medical Center; Chungbuk National University Hospital; Chungnam National University Hospital; Dong-A University Medical Center; Ehwa Womans University Medical Center; Fatima Hospital, Daegu; Gachon University Gil Medical Center; Inje University Pusan Paik Hospital; Kyungpook National University Hospital; Kwandong University College of Medicine, Myongji Hospital; National Health Insurance Corporation Ilsan Hospital; National Medical Center; Pusan National University Hospital; Samsung Medical Center, Seoul; Seoul Metropolitan Government, Seoul National University, Boramae Medical Center; Seoul National University Hospital; Seoul National University, Bundang Hospital; Yeungnam University Medical Center; Yonsei University, Severance Hospital; Yonsei University, Gangnam Severance Hospital; Ulsan University Hospital; Wonju Christian Hospital (in alphabetical order)]. All clinical investigations were conducted in accordance with the guidelines of the 2008 Declaration of Helsinki.

### Follow-up and outcome ascertainment

The follow-up period was May 2009–December 2013, and patients were followed up for the occurrence of death. Mortality was classified as all-cause, cardiovascular, or infection-related. Cardiovascular mortality was defined as death from myocardial infarction, congestive heart failure, arrhythmia, cerebrovascular disease, or sudden death. Infection-related mortality was defined as death from pneumonia, abdominal infection, endocarditis, central nervous system infection, genitourinary infection, or septicemia. Others included liver failure, gastro-intestinal hemorrhage, diabetic coma, cachexia/failure to thrive, air embolism, withdrawal from dialysis, accident unrelated to treatment, and suicide. The information regarding death was classified based on electronic medical records and above criteria, reported, reviewed, and confirmed by physicians responsible for conducting the report.

### Selection of conventional risk factors and multiple inflammatory markers

Age, sex, body mass index (BMI), systolic blood pressure (BP), diastolic BP, presence of diabetes, history of MI, smoking, use of anti-hypertensive agents, total cholesterol, low-density lipoprotein (LDL), high-density lipoprotein (HDL), and dialysis vintage were selected as conventional risk factors for mortality. For patients on hemodialysis (HD) and peritoneal dialysis (PD), pre-dialysis BP at the HD center and BP at the PD clinic measured with automatic devices in the sitting position after resting for at least 5 minutes were used for analysis, respectively. Inflammatory markers, often measured in clinical practice, including serum albumin, high-sensitivity C-reactive protein (hsCRP), WBC, and ferritin were obtained at the time of enrollment in this prospective cohort.

### Statistical analysis

Data were expressed as mean ± standard deviation or medians with ranges. Differences between groups were analyzed by Student’s t-tests and chi-squared tests as appropriate. Skewed distribution variables (hsCRP, ferritin) were log-transformed to attain normal distribution. Hazard ratios were calculated with Cox proportional hazard models to adjust for confounding factors. Age, sex, BMI, systolic BP, diastolic BP, presence of diabetes, history of MI, smoking, use of anti-hypertensive agents, total cholesterol, LDL, HDL, and dialysis vintage were considered as possible confounders.

The NRI and IDI were calculated to evaluate the added predictive ability of the inflammatory markers beyond that of the conventional risk factors. Individual inflammatory markers or different combinations of inflammatory markers were added to a model with established risk factors. Time-dependent receiver operating characteristic (ROC) curves were constructed to determine the additional predictive value of each inflammatory marker and its combination. Cox proportional hazards regression analysis was used to derive a prediction model of all-cause mortality. Model 1 included all conventional risk factors; Model 2 included only statistically significant conventional risk factors and inflammatory markers, which were selected by the backward elimination method; Model 3 consisted of conventional risk factors and a combination of inflammatory markers with the highest NRI value. Models 1, 2, and 3 were reconstructed according to dialysis modality. The integrated area under the curve (iAUC) was calculated to compare the predictive accuracy of the models. The iAUC is a weighted average of the AUC over a follow-up period and a measure of predictive accuracy of the model during follow-up, with a higher iAUC indicating a better predictive accuracy. Differences in iAUC between the models were calculated using a bootstrapping method with resampling of 1000 times. The estimated difference in iAUC was used to compare the accuracy among the predictive models. The 1-, 3-, and 5-year mortality risk calculator was constructed using Model 3.

The statistical analysis was performed using SAS system for Windows, version 9.2 (SAS Institute Inc., Cary, NC) and R (R Foundation for Statistical Computing, Vienna, Austria; www.r-project.org). P values < 0.05 were considered statistically significant.

## Results

### Baseline characteristics

Table 1 shows patients’ demographic, clinical, and biochemical characteristics according to dialysis modality. The mean age of the subjects was 61.7 years, and 58.8% were men. Diabetes was the most common cause of ESRD (51.6%). Patients on PD were younger and had a lower proportion of diabetes, coronary artery disease, cerebrovascular disease, and smoking than patients on HD had.

**Table 1:** Baseline demographic, clinical, and biochemical characteristics according to dialysis modality.

	Entire (n = 3,309)	HD (n = 2,356)	PD (n = 953)	P value
**Age (year)**	61.7 ± 13.6	63.6 ± 13.5	57.2 ± 12.8	< 0.001
**Male sex, n (%)**	1946 (58.8)	1388 (58.9)	558 (58.6)	0.85
**Duration of dialysis (months)**	59.4 ± 46.1	59.7 ± 48.2	58.7 ± 40.4	0.56
**Follow-up duration (months)**	35.0 ± 15.6	34.5 ± 15.4	36.0 ± 16.2	0.02
**Body mass index (kg/m^2^)**	22.7 ± 3.4	22.6 ± 3.4	23.2 ± 3.3	< 0.001
**Blood pressure (mmHg)**				
**Systolic BP**	140.8 ± 22.0	143.2 ± 21.9	134.8 ± 21.1	< 0.001
**Diastolic BP**	77.7 ± 13.4	77.0 ± 13.5	79.3 ± 13.0	< 0.001
**Diabetes, n (%)**	1710 (51.7)	1281 (54.4)	429 (45.0)	< 0.001
**History of MI, n (%)**	474 (14.3)	375 (15.9)	99 (10.4)	< 0.001
**Current smoking, n (%)**	329 (9.9)	246 (10.4)	83 (8.7)	0.13
**Use of anti-HTN therapy, n (%)**				
**ARB or ACEi**	1878 (56.8)	1249 (33.0)	629 (66.0)	< 0.001
**β-blocker**	1646 (49.7)	1131 (48.0)	515 (54.0)	0.002
**Others**	2681 (81.0)	1855 (78.7)	826 (86.7)	< 0.001
**Other comorbidity, n (%)**				
**Malignancy**	220 (6.7)	189 (8.0)	31 (3.3)	< 0.001
**Cerebrovascular disease**	300 (9.1)	232 (9.9)	68 (7.1)	0.01
**Other cardiovascular disease** **(CHF, arrhythmia)**	516 (15.6)	373 (15.8)	143 (15.0)	0.55
**Cause of ESRD, n (%)**				
**Diabetes**	1710 (51.6)	1281 (54.4)	429 (45.0)	< 0.001
**Hypertension**	575 (17.4)	385 (16.3)	190 (20.0)	
**Glomerulonephritis**	431 (13.0)	256 (10.9)	175 (18.3)	
**Others**	593 (18.0)	434 (18.4)	159 (16.7)	
**Lipid profile, mg/dL**				
**Total Cholesterol**	158.5 ± 43.3	153.9 ± 41.8	170.1 ± 44.8	< 0.001
**LDL**	89.2 ± 33.8	85.3 ± 32.4	99.5 ± 35.3	< 0.001
**HDL**	41.0 ± 13.4	40.8 ± 13.3	41.5 ± 13.6	0.18
**Serum inflammatory markers**				
**Ferritin, median (interquartile range), ng/mL**	197.2 (100.9–365.6)	204.9 (110.3–371.9)	179.0 (82.5–350.0)	< 0.001
**WBC, mean ± SD, ×10^3^/μL**	6.736 ± 2.597	6.665 ± 2.637	6.912 ± 2.488	0.01
**hsCRP, median (interquartile range), mg/L**	0.2 (0–1.1)	0.3 (0.1–1.4)	0.1 (0.0–0.6)	< 0.001
**Albumin, mean ± SD, g/dL**	3.6 ± 0.6	3.6 ± 0.6	3.6 ± 0.6	< 0.001

Values are shown as mean ± standard deviation or median with interquartile range.

Abbreviations: ACEi, angiotensin-converting enzyme inhibitor; ARB, angiotensin receptor blocker; BP, blood pressure; CHF, congestive heart failure; ESRD, end stage renal disease; HD, hemodialysis; HDL, high density lipoprotein; hsCRP, high-sensitivity C-reactive protein; HTN, hypertension; LDL, low density lipoprotein; MI, myocardial infarction; PD, peritoneal dialysis; WBC, white cell count

### Causes of death

During the mean follow-up of 2.9 years, 661 (20.0%) deaths occurred. Cardiovascular disease (37.0%) was the most common cause of death, followed by infection (27.1%) (Table 2).

**Table 2:** Causes of death of patients on dialysis according to dialysis modality.

	Entire (n = 3,309)	HD (n = 2,356)	PD (n = 953)
**Cardiovascular disease, n (%)**	244 (37.0)	163 (34.7)	81 (42.4)
**Cardiovascular disease**	92 (13.9)	67 (14.3)	25 (13.1)
**Cerebrovascular disease**	41 (6.2)	31 (6.6)	10 (5.2)
**Sudden death**	111 (16.8)	65 (13.8)	46 (24.1)
**Infectious disease, n (%)**	179 (27.1)	108 (23.0)	71 (37.2)
**Cancer, n (%)**	37 (5.6)	33 (7.0)	4 (2.1)
**Other, n (%)**	74 (11.2)	58 (12.3)	16 (8.4)
**Unknown, n (%)**	127 (19.2)	108 (23.0)	19 (9.9)
**Total, n (%)**	661 (100.0)	470 (100.0)	191 (100.0)

Abbreviations: HD, hemodialysis; PD, peritoneal dialysis

### Association of individual inflammatory markers with all-cause, cardiovascular, and infection-related mortalities: Cox proportional hazard models

Serum albumin, hsCRP, and WBC were independent predictors of all-cause, cardiovascular, and infection-related mortalities after adjusting confounding factors in the entire population (all P < 0.05) (Table 3). In patients receiving HD, serum albumin and hsCRP were independent predictors of all-cause, cardiovascular, and infection-related mortalities (all P < 0.05). Serum WBC was also an independent predictor of all-cause death (P = 0.001) in patients on HD. In patients undergoing PD, serum albumin, hsCRP, and WBC were independent predictors of all-cause and infection-related mortalities (all P < 0.05). Serum ferritin only showed a significant association with all-cause mortality in patients on PD (P = 0.03).

**Table 3:** Multivariate adjusted hazard ratios in all-cause, cardiovascular, and infection-related mortality.

	All-cause		Cardiovascular		Infection	
	HR* (95% Cl)	P value	HR* (95% Cl)	P value	HR* (95% Cl)	P value
**Entire**						
**Ferritin**	1.09 (0.99–1.20)	0.08	1.01 (0.87–1.18)	0.90	1.04 (0.87–1.24)	0.71
**WBC**	1.08 (1.04–1.12)	**< 0.001**	1.06 (1.00–1.13)	**0.04**	1.11 (1.04–1.18)	**0.001**
**hsCRP**	1.12 (1.07–1.18)	**< 0.001**	1.09 (1.01–1.17)	**0.03**	1.23 (1.13–1.34)	**< 0.001**
**Albumin**	0.52 (0.45–0.60)	**< 0.001**	0.64 (0.50–0.83)	**< 0.001**	0.39 (0.30–0.50)	**< 0.001**
**HD**						
**Ferritin**	1.10 (0.98–1.23)	0.13	0.98 (0.82–1.18)	0.86	1.00 (0.79–1.27)	0.99
**WBC**	1.07 (1.03–1.11)	**0.001**	1.06 (1.00–1.14)	0.07	1.07 (0.99–1.17)	0.10
**hsCRP**	1.11 (1.05–1.17)	**< 0.001**	1.10 (1.01–1.20)	**0.026**	1.19 (1.06–1.32)	**0.002**
**Albumin**	0.54 (0.45–0.64)	**< 0.001**	0.69 (0.50–0.95)	**0.021**	0.40 (0.29–0.54)	**< 0.001**
**PD**						
**Ferritin**	1.22 (1.02–1.45)	**0.03**	1.15 (0.87–1.52)	0.34	1.32 (1.00–1.74)	0.05
**WBC**	1.12 (0.03–1.22)	**0.008**	1.07 (0.92–1.23)	0.39	1.19 (1.06–1.33)	**0.003**
**hsCRP**	1.23 (1.10–1.37)	**< 0.001**	1.04 (0.86–1.25)	0.71	1.40 (1.20–1.64)	**< 0.001**
**Albumin**	0.56 (0.41–0.78)	**< 0.001**	0.72 (0.42–1.21)	0.21	0.49 (0.30–0.80)	**0.004**

*Adjusted for conventional risk factors: age, sex, BMI, systolic BP, diastolic BP, diabetes, history of MI, smoking, use of anti-HTN therapy, LDL, HDL, total cholesterol, dialysis vintage

Abbreviations: BMI, body mass index; BP, blood pressure; CI, confidence interval; HD, hemodialysis; HDL, high density lipoprotein; HR, hazard ratio; hsCRP, high-sensitivity C-reactive protein; HTN, hypertension; LDL, low density lipoprotein; MI, myocardial infarction; PD, peritoneal dialysis; WBC, white cell count

### Incremental predictive ability of multiple inflammatory makers for all-cause, cardiovascular, and infection-related mortalities according to dialysis modality: NRI, IDI

Table 4 shows the additional predictive powers of individual inflammatory markers for cause-specific mortalities in patients on dialysis according to dialysis modality. When added to the model with conventional risk factors, albumin reclassified 17.2% (P < 0.001), hsCRP 15.7% (P < 0.001), and WBC 6.8% (P = 0.02) of patients, resulting in better all-cause mortality risk prediction in the entire population. In the entire population, the NRI for the addition of the combination of inflammatory markers and conventional risk factors was highest for the combination of serum albumin, hsCRP, WBC, and ferritin for all-cause mortality (21.0%, P < 0.001) and for the combination of serum albumin, hsCRP, and WBC for cardiovascular mortality (21.3%, P < 0.001).

**Table 4:** Predictive power of individual and multiple biomarker models for all-cause, cardiovascular, and infection-related mortality.

	All-cause		Cardiovascular		Infection	
	NRI	IDI	NRI	IDI	NRI	IDI
**Entire**						
*CR–Ferritin	6.6	0.002	2.5	0.000	3.8	0.000
*CR–WBC	**6.8^†^**	**0.010^‡^**	3.1	0.003	7.9	**0.007^†^**
*CR–hsCRP	**15.7^‡^**	**0.013^‡^**	**15.1^†^**	**0.006^†^**	**22.8^‡^**	**0.015^‡^**
*CR–Albumin	**17.2^‡^**	**0.033^‡^**	**15.2^†^**	**0.007^†^**	**28.5^‡^**	**0.039^‡^**
*CR–Albumin + hsCRP	**19.2^‡^**	**0.040^‡^**	**20.4^‡^**	**0.010^‡^**	**24.0^‡^**	**0.051^‡^**
*CR–Albumin + hsCRP + WBC	**20.8^‡^**	**0.045^‡^**	**21.3^‡^**	**0.011^‡^**	**28.1^‡^**	**0.054^‡^**
*CR–All biomarkers	**21.0^‡^**	**0.045^‡^**	**20.7^†^**	**0.012^‡^**	**26.8^‡^**	**0.055^‡^**
**HD**						
*CR–Ferritin	9.1	0.001	-4.7	0.000	3.1	0.000
*CR–WBC	4.3	0.008^†^	-1.1	0.003	9.5	0.004
*CR–hsCRP	**14.5^†^**	**0.012^‡^**	**17.9^†^**	**0.007^†^**	**21.9^†^**	**0.010^†^**
*CR–Albumin	**14.2^‡^**	**0.028^‡^**	11.8	**0.006^†^**	**23.9^†^**	**0.040^‡^**
*CR–Albumin + hsCRP	**19.0^‡^**	**0.035^‡^**	**18.5^†^**	**0.011^†^**	**24.7^‡^**	**0.047^‡^**
*CR–Albumin + hsCRP + WBC	**16.8^‡^**	**0.039^‡^**	**15.4^†^**	**0.012^†^**	**25.7^‡^**	**0.049^‡^**
*CR–All biomarkers	**17.3^‡^**	**0.039^‡^**	**14.3^†^**	**0.014^‡^**	**27.1^‡^**	**0.053^‡^**
**PD**						
*CR–Ferritin	11.2	0.014	8.7	0.008	10.8	0.013
*CR–WBC	12.9	0.019^†^	16.0	0.003	7.5	0.033
*CR–hsCRP	**22.3^†^**	**0.025^†^**	11.3	0.002	**24.8^†^**	**0.057^†^**
*CR–Albumin	16.0	**0.022^†^**	13.3	0.006	21.6	0.027
*CR–Albumin + hsCRP	**20.7^†^**	**0.039^†^**	10.6	0.006	**28.4^†^**	**0.072^†^**
*CR–Albumin + hsCRP + WBC	**25.2^†^**	**0.049^†^**	13.3	0.008	**26.4^‡^**	**0.089^‡^**
*CR–All biomarkers	**25.7^†^**	**0.061^†^**	18.5	0.014	**25.9^†^**	**0.100^‡^**

*CR, conventional risk factor, i.e., age, sex, BMI, systolic BP, diastolic BP, diabetes, history of MI, smoking, use of anti-HTN therapy, LDL, HDL, total cholesterol, dialysis vintage

^†^P < 0.05

^‡^P < 0.001

Abbreviations: BMI, body mass index; BP, blood pressure; CI, confidence interval; HD, hemodialysis; HDL, high density lipoprotein; HR, hazard ratio; hsCRP, high-sensitivity C-reactive protein; HTN, hypertension; IDI, integrated discrimination improvement; LDL, low density lipoprotein; MI, myocardial infarction; NRI, net reclassification improvement; PD, peritoneal dialysis; WBC, white cell count

In patients on HD, the NRI for the incremental combination of inflammatory markers and conventional risk factors was maximized for the combination of serum albumin and hsCRP for all-cause (19.0%, P < 0.001) and cardiovascular mortalities (18.5%, P = 0.02) and for the combination of serum albumin, hsCRP, WBC, and ferritin for infection-related mortality (27.1%, P < 0.001).

In patients on PD, the NRI for the addition of the combination of inflammatory markers and the conventional risk factors was highest for the combination of serum albumin, hsCRP, WBC, and ferritin for all-cause (25.7%, P = 0.004) and for the combination of serum albumin and hsCRP for infection-related mortality (28.4%, P = 0.004). No significant incremental predictive values of the inflammatory markers beyond the standard risk factors alone for cardiovascular mortality were found in patients on PD. Discrimination analysis for significant inflammatory markers also confirmed the results of the reclassification analysis.

### Best prediction model for all-cause mortality according to dialysis modality

**Fig 1:**
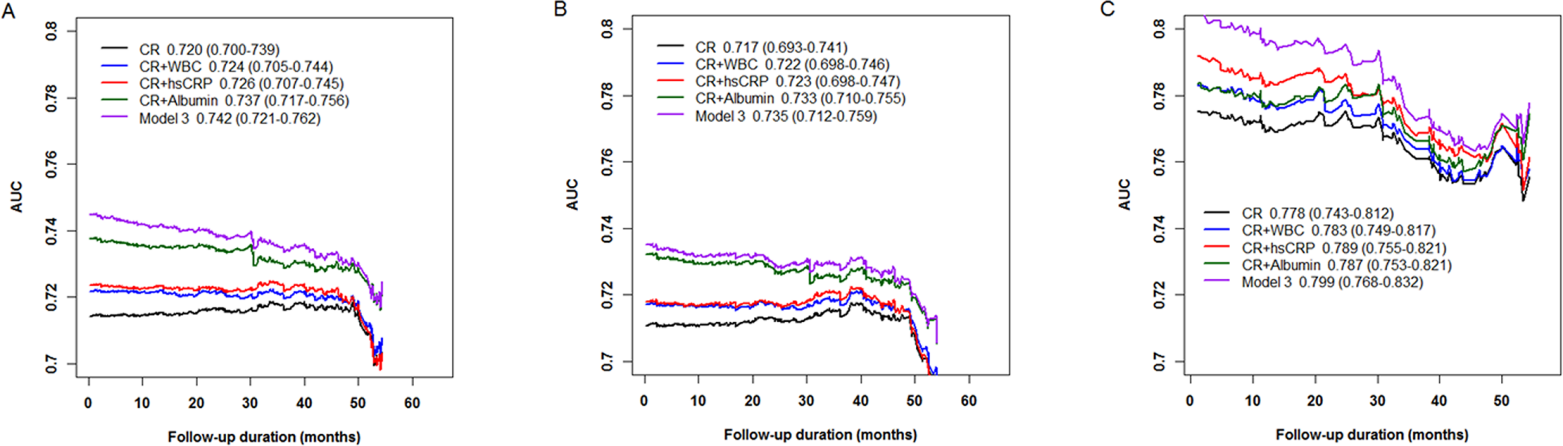
Time-dependent receiver operating characteristic curves for all-cause mortality for patients on dialysis according to dialysis modality. In the entire population (A), iAUC values for all-cause mortality were 0.720 (95% CI, 0.700–0.739) for the crude model, including conventional risk factors, 0.724 (95% CI, 0.705–0.744) for the crude model plus WBC, 0.726 (95% CI, 0.707–0.745) for the crude model plus hsCRP, 0.737 (95% CI, 0.717–0.756) for the crude model plus albumin, 0.742 (95% CI, 0.721–0.762) for the crude model plus albumin, hsCRP, WBC, and ferritin. The differences in iAUC were -0.0046 (-0.010 to -0.001) for WBC, -0.006 (-0.012 to -0.002) for hsCRP, -0.017 (-0.028 to -0.009) for albumin, -0.022 (95% CI, -0.033 to -0.013) for albumin, hsCRP, WBC, and ferritin, demonstrating that although individual inflammatory markers significantly improved the predictive accuracy for all-cause mortality, the integration of all inflammatory markers resulted in the most accurate prediction model. In patients on HD (B), iAUC values for all-cause mortality were 0.717 (95% CI, 0.693–0.741) for the crude model, 0.722 (95% CI, 0.698–0.743) for the crude model plus WBC, 0.723 (95% CI, 0.698–0.747) for the crude model plus hsCRP, 0.733 (95% CI, 0.710–0.755) for the crude model plus albumin, 0.735 (95% CI, 0.712–0.759) for the crude model plus albumin, hsCRP, WBC, and ferritin. The differences in iAUC were -0.0043 (-0.010 to -0.0004) for WBC, -0.005 (-0.012 to -0.0006) for hsCRP, -0.015 (-0.027 to -0.007) for albumin, -0.018 (95% CI, -0.030 to -0.008) for albumin, hsCRP, WBC, and ferritin. In patients on PD (C), iAUC values for all-cause mortality were 0.778 (95% CI, 0.743–0.812) for the crude model, 0.783 (95% CI, 0.749–0.817) for the crude model plus WBC, 0.789 (95% CI, 0.755–0.821) for the crude model plus hsCRP, 0.787 (95% CI, 0.753–0.821) for the crude model plus albumin, 0.799 (95% CI, 0.768–0.832) for the crude model plus albumin, hsCRP, WBC, and ferritin. The differences in iAUC were -0.004 (-0.015 to 0.0003) for WBC, -0.010 (-0.025 to -0.002) for hsCRP, -0.009 (-0.023 to -0.0003) for albumin, -0.021 (95% CI, -0.038 to -0.007) for albumin, hsCRP, WBC, and ferritin.

Time-dependent ROC curves are presented in Fig 1. In the entire population, iAUC values for all-cause mortality were 0.720 (95% CI, 0.700–0.739) for the basic model, including conventional risk factors, 0.724 (95% CI, 0.705–0.744) for the basic model plus WBC, 0.726 (95% CI, 0.707–0.745) for the basic model plus hsCRP, 0.737 (95% CI, 0.717–0.756) for the basic model plus albumin, 0.742 (95% CI, 0.721–0.762) for the basic model plus albumin, hsCRP, WBC, and ferritin. The differences in iAUC were -0.0046 (-0.010 to -0.001) for WBC, -0.006 (-0.012 to -0.002) for hsCRP, -0.017 (-0.028 to -0.009) for albumin, -0.022 (95% CI, -0.033 to -0.013) for albumin, hsCRP, WBC, and ferritin, demonstrating that although each inflammatory marker significantly improved the predictive accuracy for all-cause mortality, the combination of all inflammatory markers resulted in the most accurate prediction model. The results of time-dependent ROC curves according to dialysis modality showed the most consistent result (Fig 1B and 1C).

The hazard ratios for the variables and iAUC and estimated difference in iAUC for the models of all-cause mortality are shown in Table 5. iAUC values were 0.719 (95% CI, 0.700–0.738) for Model 1, with conventional risk factors, 0.734 (95% CI, 0.714–0.754) for Model 2, with only significant conventional risk factors and inflammatory markers, 0.741 (95% CI, 0.722–0.761) for Model 3, with conventional risk factors and the combination of inflammatory markers with the highest NRI value. The difference in iAUC between Models 1 and 2 was -0.015 (95% CI, -0.027 to -0.005), and the difference between Models 1 and 3 was -0.022 (95% CI, -0.033 to -0.013), indicating that there was significant improvement in predictive accuracy between models. Reconstructed predictive models according to dialysis modality showed consistent results.

**Table 5:** Hazard ratios of each variable and comparing the predictive accuracy of the models.

	Entire			HD			PD		
	Model 1	Model 2	Model 3	Model 1	Model 2	Model 3	Model 1	Model 2	Model 3
**Age**	**1.060**^†^	**1.057**^†^	**1.059**^†^	**1.060**^†^	**1.058**^†^	**1.059**^†^	**1.076**^†^	**1.067**^†^	**1.066**^†^
**Sex (female)**	0.906		0.876	0.857	0.790*	0.846	1.257		1.191
**BMI**	0.970*		0.976	0.955*	0.954*	0.960*	1.008		1.035
**Systolic BP**	0.996		0.996	0.999		0.998	1.001		0.999
**Diastolic BP**	1.005		1.007	1.005		1.008	0.995		0.994
**Diabetes**	**1.663**^†^	**1.468**^†^	**1.552**^†^	**1.574**^†^	1.366*	**1.502**^†^	1.908*	**1.976**^†^	1.916*
**History of MI**	1.322*	1.356*	1.349*	1.329*	1.390*	**1.393**^†^	1.275		1.137
**Smoking**	1.278		1.152	1.405*		1.321	0.999		1.172
**ARB or ACEi**	0.926		0.896	0.911		0.871	0.695		0.812
**β-blocker**	1.060		1.017	0.999		0.988	1.235		1.186
**Other anti-HTN therapy**	0.963		1.034	0.949		1.002	0.837		0.853
**Total cholesterol**	0.999		0.999	0.998		0.999	1.001		1.002
**LDL**	1.000		1.000	0.999		0.998	0.995		0.995
**HDL**	1.003		1.005	1.003		1.004	1.003		1.012
**Dialysis vintage**	1.002	**1.004**^†^	**1.004**^†^	0.999		1.003	**1.008**^†^	**1.011**^†^	**1.012**^†^
**Ferritin**			1.027					1.194*	1.209*
**WBC**		1.046*	**1.049**^**‡**^						1.074
**hsCRP**		1.062*	1.061*		1.052*	1.067*		**1.192**^†^	1.173*
**Albumin**		**0.549**^†^	**0.548**^†^		**0.577**^†^	**0.562**^†^		0.657*	0.645*
**iAUC (95% CI)**	0.719 (0.700–0.738)	0.734 (0.714–0.754)	0.741 (0.722–0.761)	0.718 (0.693–0.741)	0.727 (0.702–0.749)	0.736 (0.711–0.759)	0.779 (0.747–0.811)	0.784 (0.754–0.812)	0.799 (0.769–0.829)
**Estimated difference in iAUC (95% CI)**		-0.015 (-0.027– -0.005)	-0.022 (-0.033– -0.013)		-0.009 (-0.021– -0.001)	-0.018 (-0.030– -0.008)		-0.005 (-0.025– -0.013)	-0.021 (-0.039– -0.007)

*P < 0.05,

^†^P < 0.001

Abbreviations: ACEi, angiotensin-converting enzyme inhibitor; ARB, angiotensin receptor blocker; BMI, body mass index; BP, blood pressure; CI, confidence interval; HD, hemodialysis; HDL, high density lipoprotein; HR, hazard ratio; hsCRP, high-sensitivity C-reactive protein; HTN, hypertension; iAUC, integrated area under the curve; LDL, low density lipoprotein; MI, myocardial infarction; PD, peritoneal dialysis; WBC, white cell count

The mortality risk calculator (S1 Fig) and prediction equations (S2 Fig) based on Model 3 are presented in the supplementary data. This calculator provides 1-, 3-, and 5-year mortality rates in patients with ESRD according to dialysis modality.

## Discussion

This prospective multicenter study, with 3,309 patients on dialysis, demonstrated that multi-marker approaches using a combination of multiple inflammatory markers (serum albumin, hsCRP, WBC, and ferritin) practically available in a clinical setting, improve the predictive ability for all-cause mortality beyond established risk factors, as measured by improvements in the NRI, IDI, and C-statistic estimate (iAUC). Additionally, we found that optimal risk stratification for cause-specific mortality could be accomplished by different combinations of inflammatory markers according to dialysis modality. Based on this result, we have developed the best risk prediction models for all-cause mortality according to dialysis modality by adding a combination of serum albumin and hsCRP in patients on HD, and serum albumin, hsCRP, WBC, and ferritin in patients on PD to a crude model including the Framingham risk score [2]. To our best knowledge, this is the first report that constructs predictive models for long-term as well as short-term mortality in patients with ESRD by using easily available multiple inflammatory markers with the greatest predictability assessed by reclassification statistics.

Despite numerous studies regarding the association between each inflammatory marker and clinical outcomes [7–16], the question regarding incremental predictabilities for mortality by combining significant inflammatory markers in patients with ESRD remains uncertain. In this study, we tried to address the question of whether the incremental combination of the serum markers of inflammation, like cardiac biomarkers, could increase the mortality prediction in patients on dialysis. One of the principal findings of our study was that the addition of any combination of inflammatory markers (albumin plus hsCRP, albumin plus hsCRP plus WBC, albumin plus hsCRP plus WBC plus ferritin) to the crude model significantly enhanced the prediction for all-cause mortality with a somewhat different degree of improvement irrespective of dialysis modality. Serum albumin, hsCRP, WBC, and ferritin individually might reflect a different pathophysiological pathway on cardiovascular, infection-related, or other mortalities. Thus, simultaneous measurement of these four biomarkers led to complementary prognostic information about survival. Notably, among these combinations of inflammatory markers, the combination of albumin and hsCRP in patients on HD and albumin, hsCRP, WBC, and ferritin in patients on PD, which was based on the NRI results, yielded the greatest predictive power for all-cause mortality. This differing prognostic information, according to dialysis modality, could provide different clinical monitoring and decision making by nephrologists and better information regarding prognosis to patients with ESRD and their families.

Prediction models for all-cause mortality were constructed by using a combination of inflammatory markers with the highest NRI values according to dialysis modality. It was not anticipated that the model with the addition of inflammatory markers with the highest NRI values to the crude model (Model 3) would be more accurate than both the model including only significant clinical and laboratory variables (Model 2) and the crude model (Model 1). This means that a combination of only the significant indicators does not ensure the most superior prediction model for mortality. Considering that heterogeneous pathological conditions contribute to the deaths of patients with ESRD, although not all traditional risk factors are significant, the null model harboring Framingham risk factors, plus the most predictable combination of inflammatory markers, yielded the best risk models for all-cause deaths. Thus, our findings have significance, since existing mortality prediction models in ESRD have mainly selected only significant variables identified by logistic regression and used a calculation of a point score to draw risk prediction models [1,20–22]. Our risk prediction models using easily accessible clinical, demographic, and laboratory data could be used by clinicians to distinguish patients on dialysis, who are high risk, and to make decisions regarding further clinical evaluations and timely interventions. Moreover, mortality prediction models according to dialysis modality enable a more individualized outcome prediction in patients on dialysis and thus improve patient survival.

Another notable finding of this study was that the simultaneous addition of inflammatory markers to established risk factors improved risk stratification for death from infectious causes regardless of dialysis modality and cardiovascular mortality in patients on HD. In particular, the best combination of inflammatory markers for predicting cause-specific mortality was different according to dialysis modality. The combination of albumin and CRP measurements resulted in the most improved predictive power for cardiovascular death in patients on HD and for infection-related death in patients on PD. However, risk prediction for cardiovascular mortality was not improved with the addition of any combination of inflammatory markers or each inflammatory marker alone, compared to conventional risk factors in patients on PD. This interesting result could be partly explained by the differential predictive value of serum albumin for cardiovascular and infection-related mortality according to dialysis modality. One study of 130,052 patients on dialysis (HD, 117,851; PD, 12,171) demonstrated that, despite having the same level of serum albumin, the adjusted risk of cardiovascular death for patients on PD was lower than for that for patients on HD, and the risk of infection-related death was higher in patients on PD than patients on HD [7]. Patients undergoing PD experience daily peritoneal protein loss, which is predominantly albumin [23]. Our study and previous study suggested that decreased serum albumin levels associated with peritoneal albumin losses may not increase mortality, especially cardiovascular death, for patients on PD. Considering the relatively slight clinical significance of increased inflammatory markers beyond the conventional cardiovascular risk factors on cardiovascular mortality in patients on PD, clinicians should focus on treating and improving the Framingham risk factors of patients undergoing PD, rather than monitoring inflammatory markers to reduce cardiovascular death.

This study has several limitations. First, we could not confirm the results using a different validation cohort. A validation cohort is needed to confirm the utility of risk prediction models using inflammatory markers for mortality according to dialysis modality. Second, we only analyzed inflammatory markers at the time of enrollment. Considering that the systemic inflammatory response has intraindividual variability over time in patients on dialysis [24], further studies using serial measurement and analyzing the variation of inflammatory markers are needed. Third, this study only included patients with ESRD in Korea. Previous studies have reported that patients on dialysis in Asian countries have a lower degree of inflammation compared with those in the United States and Europe [9,25,26]. This difference requires caution in generalizing our results across race and ethnicity. Finally, we could not clearly explain the underlying mechanism driving the difference of the optimal combination of inflammatory markers between dialysis modalities. Further study is needed to clarify a cause-and-effect relationship.

Nevertheless, our study has important strengths. Our predictive models for mortality are practical, in that all the variables are routinely measured in patients with ESRD and could be simply implemented in real-world clinics. The inflammatory markers we used are readily and easily measureable parameters compared with pro-inflammatory cytokines. Another crucial implication of this study is that different combinations of inflammatory markers, according to dialysis modality, yielded different best predictive values for cause-specific mortality. Considering that both differentiating between patients on dialysis at high and low cause-specific mortality risk and clinical decision making in patients with ESRD are challenging, this detailed result enables a stratified risk assessment in this population according to dialysis modality. Furthermore, this study provides evidence that properly selecting the number of inflammatory markers and the correct follow-up period according to dialysis modality and cause-specific mortality, could contribute to increasing cost-effectiveness, as well as to improving patient survival. Finally, this current report has attempted for the first time to merge reclassification statistics into risk prediction models and proved superiority in accuracy of risk models.

In summary, we found that multi-marker approaches using multiple inflammatory markers practically available in clinics have additional predictive value for all-cause mortality in patients on dialysis, and we developed highly accurate predictive models for mortality, according to dialysis modality, using routine inflammatory laboratory data. Our accurate risk calculator for prediction of mortality could enable individualized decision-making and result in early and appropriate therapies and preventions, which could improve outcomes of patients undergoing dialysis and might reduce the burdens of ESRD.

## Supporting information

S1 Fig Mortality risk calculator. This calculator shows 1-, 3-, and 5-year mortality rates in patients with ESRD according to dialysis modality.
(XLS)

S2 Fig Prediction equations for the 3-year risk of death. Mortality prediction equations are provided according to dialysis modality.
(DOCX)
