# Supplementary material for: Individualized prediction of mortality using multiple inflammatory markers in patients on dialysis
Source: PLoS One. 2018 Mar 1;13(3):e0193511. doi: 10.1371/journal.pone.0193511 (PMC5832435; doi:10.1371/journal.pone.0193511)
Supplement: S2 Fig — Mortality prediction equations are provided according to dialysis modality. (DOCX) [file pone.0193511.s002.docx]

**Prediction equations for the 3-year risk of death.**

Entire

P=1-0.897 ^ exp (0.057×(Age-61.744)-0.132×(Sex-1.412)-0.025×(BMI-22.732)-0.004×(SBP-140.802)+0.007×(DBP-77.689)+0.439×(DM-0.517)

+0.300×(CAD-0.143)+0.142×(Smoking-0.099) -0.109×(ARB or ACEi -0.568)+0.016×(β-blocker -0.497)+0.034×(other drug -0.810)

-0.0005×(LDL-89.230)+0.005×(HDL-41.001)-0.001×(Total Cholesterol -158.549) +0.004×(Dialysis vintage -24.873)

+0.059×(ln(hs-CRP)+1.166)+0.027×(ln(Ferritin)-5.217)+0.048×(WBC/1000-6.736)-0.601×(Albumin-3.624))

HD

P=1-0.885 ^ exp (0.057×(Age-63.597)-0.167×(Sex-1.411)-0.041×(BMI-22.560)-0.002×(SBP-143.156)+0.008×(DBP-77.049)+0.407×(DM-0.544)

+0.332×(CAD-0.159)+0.278×(Smoking-0.104)-0.138×(ARB or ACEi -0.530)-0.012×(β-blocker -0.480)+0.002×(other drug -0.787)

-0.002×(LDL-85.260)+0.004×(HDL-40.797)-0.001×(Total Cholesterol -153.889+0.002×(Dialysis vintage -25.552))

+0.065×(ln(hs-CRP)+1.016)-0.577×(Albumin-3.648))

PD

P=1-0.925 ^ exp (0.064×(Age-57.162)+0.175×(Sex-1.414)+0.034×(BMI-23.170)-0.001×(SBP-134.849)-0.006×(DBP-79.310)+0.650×(DM-0.450)

+0.128×(CAD-0.104)+0.159×(Smoking-0.087)-0.208×(ARB or ACEi -0.660)+0.170×(β-blocker -0.540)-0.159×(other drug -0.867)

-0.005×(LDL-99.495)+0.012×(HDL-41.509)+0.002×(Total Cholesterol -170.053)+0.012×(Dialysis vintage -23.162)

+0.160×(ln(hs-CRP)+1.537)+0.190×(ln(Ferritin)-5.102)+0.071×(WBC/1000-6.912)-0.439×(Albumin-3.564))
